# Characteristics and risk factors for extrapancreatic infection in patients with moderate or severe acute pancreatitis

**DOI:** 10.1016/j.heliyon.2023.e13131

**Published:** 2023-01-20

**Authors:** Tongtian Ni, Yi Wen, Bing Zhao, Ning Ning, Erzhen Chen, Enqiang Mao, Weijun Zhou

**Affiliations:** Department of Emergency, Ruijin Hospital, Shanghai Jiao Tong University School of Medicine, Shanghai, China

**Keywords:** Acute pancreatitis, Extrapancreatic infection, Risk factors, Prognosis

## Abstract

**Background:**

How to detect acute pancreatitis (AP) complicated with infection early and how to arrange the treatment time are still the main problems in the world. There are few reports on the potential relationship between extrapancreatic infections and AP. The purpose of this article was to investigate the characteristics, influencing factors and prognosis of extrapancreatic infection in AP patients with modified Marshall score ≥2 on admission.

**Materials and methods:**

We retrospectively analyzed AP admitted to emergency intensive care unit of Ruijin hospital within 72 h of onset from September 2019 to December 2021. In addition to the patients' baseline data, sites of infection and microorganisms outside the pancreas were collected. Microbial cultures were used to identify infections of the respiratory tract, blood, abdominal cavity, biliary tract, urinary tract and clostridium difficile in feces.

**Results:**

144 patients with AP were included, of which extrapancreatic infection accounted for 40.28%. C-reactive protein, procalcitonin, blood urea nitrogen, serum creatinine, oxygenation index, modified Marshall score, BISAP score and APACHE II score were significantly increased in the extrapancreatic infection group. The risk factors of extrapancreatic infection included blood urea nitrogen, Modified Marshall score and duration of mechanical ventilation. The positive rates of pathogenic bacteria in sputum culture, blood culture, ascites culture and bile culture were significantly higher than those in the 1–3 days after admission. The infection begins to worsen as early as 4–7 days after the onset of symptoms. Extrapancreatic infection is associated with pancreatic necrosis, the rate of laparotomy, length of hospital stay and in-hospital mortality.

**Conclusion:**

Our research has confirmed the need to prevent and monitor extrapancreatic infection in the early stage.

## 1 Introduction

Acute pancreatitis (AP) is a common acute abdominal disease with increasing incidence and mortality rate in the world [1,2]. Despite improvements in treatment and critical care, severe acute pancreatitis (SAP) is still associated with high mortality rates, and infection has become the most important risk factor for SAP death [1,3,4].

Prophylactic use of antibiotics to prevent secondary pancreatic infection remains controversial [5,6]. The etiological evidence of secondary necrosis in pancreatic infection is not easy to obtain in the early stage. How to identify pancreatic infections early and how to time treatments are still major problems to be solved in the clinic [5,7].

Pancreatic infection including peripancreatic infection is a common complication of acute pancreatitis. Extrapancreatic infections (EPI) refers to the infection of other organs except pancreas in AP patients during hospitalization, which is a common clinical complication of AP, and its common sites include blood, respiratory tract, urinary tract, abdominal cavity, biliary tract and surgical incision [8].

So far, the research on pancreatic infection has mainly focused on pancreatic and peripancreatic infection, but there are few reports on extrapancreatic infection of AP, while the potential relationship between the common infection site outside the pancreas and the course of pancreatitis infection has not been studied. Furthermore, data on their potential impact on prognosis are not unanimous [[9], [10], [11]].

Therefore, the purpose of this study is to analyze the characteristics, influencing factors and prognosis of extrapancreatic infection in AP patients, so as to help determine the treatment strategy of AP patients.

## 2 Methods

### 2.1 Study subjects

We retrospectively analyzed patients with moderately severe acute pancreatitis (MSAP) or SAP admitted to emergency intensive care unit (EICU) of Ruijin hospital from September 2019 to December 2021. According to the Atlanta classification and definitions, the diagnosis of acute pancreatitis requires two of the following three features [12]: (1) abdominal pain consistent with acute pancreatitis (acute onset of a persistent, severe, epigastric pain often radiating to the back); (2) serum lipase activity (or amylase activity) at least three times greater than the upper limit of normal; and (3) characteristic findings of acute pancreatitis on contrast-enhanced computed tomography (CECT) and less commonly magnetic resonance imaging (MRI) or transabdominal ultrasonography. MSAP was characterised by the presence of transient organ failure or local or systemic complications in the absence of persistent organ failure. SAP was regarded as any organ dysfunction of severity ≥2 lasting >48 h according to the modifed Marshall score.

Inclusion criteria: All patients aged 18–75 years, admitted to the EICU within 72 h of onset, diagnosed with MSAP or SAP which meeting the 2012 Atlanta diagnostic criteria for acute pancreatitis [12], with modified Marshall score ≥2 on admission. Pancreatic necrosis was observed on CECT or contrast-enhanced magnetic resonance imaging (CEMRI).

The exclusion criteria were pregnant or lactating women, malignant tumors, pancreatitis caused by Endoscopic Retrograde Cholangio-Pancreatography (ERCP), autoimmune diseases, and chronic organ dysfunction with existing heart, lung, liver, kidney, and blood systems before admission. This study was approved by Ruijin Hospital Ethics Committee affiliated to Shanghai Jiao Tong University School of Medicine and written informed consent was obtained from each patient or their family members.

### 2.2 Management of patients

After admission, patients with confirmed MSAP or SAP were treated conservatively with fluid resuscitation, and supportive treatment including ventilatory support when required [13]. Antibiotics were started on the day of admission. The initial antibiotic regimen for biliary pancreatitis is the use of carbapenems. Other types of pancreatitis use a strategy of cefoperazone plus metronidazole.

### 2.3 Microbiology

All samples were collected by the clinician, and then immediately transported to the microbiology laboratory in line with the standard procedures. Sputum and urine cultures were performed within 24 h of admission. Both tests were then performed twice a week until the SIRS response of patients had completely disappeared. Blood cultures were performed based on whether the body temperature was ≥38.5 °C. Cultures of bile and ascites were performed after the relevant invasive procedures. Clostridium difficile was detected in feces every week. The names of pathogenic bacteria and their antibiotic sensitivity patterns were recorded in the cases with positive culture results.

### 2.4 Clinical variables

The electronic medical records of all patients were scrutinized, and relevant data were obtained, including demographics information, etiology of acute pancreatitis, comorbidities, clinical evaluation (oxygenation index, systolic blood pressure) and biochemical analysis (white blood cell, C-reactive protein, procalcitonin, serum amylase, alanine aminotransferase, total bilirubin, blood urea nitrogen, serum creatinine). Data on acute pancreatitis severity, such as modified Marshall score and Bedside Index for Severity in Acute Pancreatitis (BISAP) score were registered [14]. The Acute Physiology and Chronic Health Evaluation (APACHE II) score was also calculated [15]. The prognostic indicators of pancreatitis recorded in this study include: degree of pancreatic necrosis, number of laparotomy cases, length of hospital stay, in-hospital mortality.

### 2.5 Definitions

Extrapancreatic infection was defined as ≥1 site of infection occurring in other organs of AP patients, diagnosed or excluded by multiple, multisite pathogen culture. Common sites include respiratory tract, the blood, abdominal cavity, biliary tract, urinary tract, and Clostridium difficile in feces.

Antibiotic-resistant bacterial infections were defined as: isolates not susceptible to at least one agent in three or more antimicrobial categories.

Multidrug resistant (MDR) bacteria [16]: refer to the bacteria that are usually resistant to three or more commonly used antibiotics.

Degree of pancreatic necrosis: less than 30%, 30%–50%, more than 50% [17].

### 2.6 Statistical analysis

The clinical data were analyzed by using SPSS 26.0 statistical software. Independent sample *t*-test was used to compare the mean values of extrapancreatic infection group and non-extrapancreatic infection group, whereas Chi-square test was used to compare categorical variables. Binary logistic regression was used to analyze the risk factors of extrapancreatic infection at different sites.

## 3 Results

### 3.1 Characteristics of the study population

**Fig. 1:**
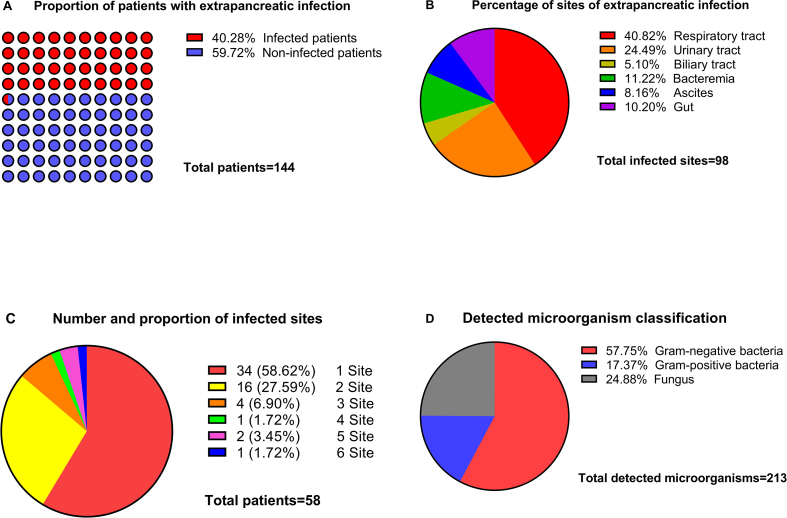
Number of infected sites and classification of detected microorganisms. (A) proportion of patients with extrapancreatic infection, (B) percentage of sites of extrapancreatic infection, (C) number and proportion of infected sites, (D) detected microorganism classification.

A total of 144 patients with acute pancreatitis with modified Marshall score ≥2 on admission aged from 17 to 75 years old were included, extrapancreatic infection accounted for 40.28% (Fig. 1A). Baseline characteristics for patients with or without extrapancreatic infection are shown in Table 1. Except for alcoholic pancreatitis, there was no significant difference in age, sex, etiology or comorbidities between the extrapancreatic infection group and the non-extrapancreatic infection group. C-reactive protein, procalcitonin, blood urea nitrogen, serum creatinine, oxygenation index, modified Marshall score, BISAP score and APACHE Ⅱ score were statistically significant between the two groups (*P* < 0.05).

**Table 1:** Comparison of baseline demographic and clinical characteristics between the extrapancreatic infection group and non-extrapancreatic infection group on admission (n%; $\bar{x}$ ±s).

	Extrapancreatic infection group (n = 58)	Non-extrapancreatic infection group (n = 86)	*P* value
Mean age (years)	45.67 ± 15.48	44.64 ± 14.20	0.680
Gender (male%)	34 (58.62%)	61 (70.93%)	0.126
Body Mass Index (kg/m^2^)	26.87 ± 3.81	26.00 ± 4.49	0.228
Etiology			
Biliary	25 (43.10%)	32 (37.21%)	0.478
Hypertriglyceridemia	31 (53.45%)	35 (40.70%)	0.132
Alcohol	0 (0.00%)	8 (9.30%)	0.021∗
Others	2 (3.45%)	11 (12.79%)	0.055
Comorbidities			
Hypertension (%)	38 (65.51%)	61 (70.93%)	0.492
Diabetes mellitus (%)	42 (72.41%)	67 (77.91%)	0.451
Indicators			
White blood cell (×10^9^/l)	12.40 ± 5.22	13.00 ± 4.92	0.484
C-reactive protein (μg/ml)	257.57 ± 118.26	206.94 ± 130.60	0.017∗tbl1fnlowast
Procalcitonin (ng/ml)	13.62 ± 30.28	4.26 ± 7.22	0.024∗tbl1fnlowast
Serum amylase (U/L)	1023.89 ± 934.00	916.43 ± 930.13	0.498
Alanine aminotransferase (U/l)	56.79 ± 91.35	66.97 ± 129.00	0.605
Total bilirubin (μmol/l)	25.33 ± 26.23	21.76 ± 17.31	0.327
Blood urea nitrogen (mmol/l)	9.13 ± 7.13	5.65 ± 3.54	0.001∗tbl1fnlowast
Serum creatinine (μmol/l)	143.45 ± 141.03	71.00 ± 38.41	<0.001∗tbl1fnlowast
Oxygenation index	216.61 ± 110.47	251.93 ± 85.80	0.033∗tbl1fnlowast
Systolic blood pressure (mmHg)	135.74 ± 23.85	139.59 ± 24.52	0.352
Modified Marshall score	3.64 ± 2.07	2.19 ± 1.05	<0.001∗tbl1fnlowast
BISAP score	2.81 ± 0.93	2.28 ± 0.52	<0.001∗tbl1fnlowast
APACHE II score	13.55 ± 5.05	9.55 ± 3.31	<0.001∗tbl1fnlowast

*Note:* BISAP score: Bedside Index for Severity in Acute Pancreatitis score, APACHE II score: Acute Physiology and Chronic Health Evaluation II score.

∗ *P* < 0.05 was considered statistically significant.

### 3.2 Characteristics of extrapancreatic infection

Among 58 patients with extrapancreatic infection, the common infection site were respiratory tract (40.82%), the urinary tract (24.49%), and the bacteremia (11.22%), and the least was biliary tract (5.10%) (Fig. 1B), and 58 patients with extrapancreatic infection combined with 1–6 sites were 34 (58.62%), 16 (27.59%), 4 (6.90%), 1 (1.72%), 2 (3.45%) and 1 (1.72%) respectively (Fig. 1C).

213 cases of pathogenic bacteria were detected. Among them, Gram-negative bacteria accounted for 58.02%, Gram-positive bacteria 17.45% and fungal infection 24.53% (Fig. 1D). The three most common pathogenic microorganisms are Acinetobacter baumannii (26.42%), Candida albicans (17.45%) and Klebsiella pneumoniae (13.21%) (Table 2).

**Table 2:** The top five pathogens of extrapancreatic infection and the drug resistance.

	Pathogenic microorganisms	n (%)	Resistant to antibiotics, n (%)		
			S	R	MDR
1	Acinetobacter baumannii	56 (26.42)	0 (0.00)	1 (1.79)	55 (98.21)
2	Candida albicans	37 (17.45)	25 (67.57)	7 (18.92)	5 (13.51)
3	Klebsiella pneumoniae	28 (13.21)	0 (0.00)	4 (14.29)	24 (85.71)
4	Pseudomonas maltophilia	16 (7.55)	14 (87.50)	2 (12.50)	0 (0.00)
5	Staphylococcus aureus	13 (6.13)	0 (0.00)	6 (46.15)	7 (53.85)
6	Other	62 (29.25)			

Note: S: sensitive to any antibiotic; R: resistant to <3 groups of antibiotics; MDR: Resistant to ≥3 groups of antibiotics.

The multidrug resistance rate of the top three Gram-negative bacteria (acinetobacter baumannii, klebsiella pneumoniae and pseudomonas maltophilia) found in extrapancreatic infections in our center was 98.21%, 85.71% and 0%, respectively. The multidrug resistance rate of common Gram-positive bacteria (staphylococcus aureus) was 53.85%. The multidrug resistance rate of common fungus (*Candida albicans*) was 13.51% (Table 2).

The culture results of respiratory tract, urinary tract, bile, blood, ascites and intestinal Clostridium difficile are listed in Table S1-S6.

### 3.3 Extrapancreatic infection and the course of disease

**Fig. 2:**
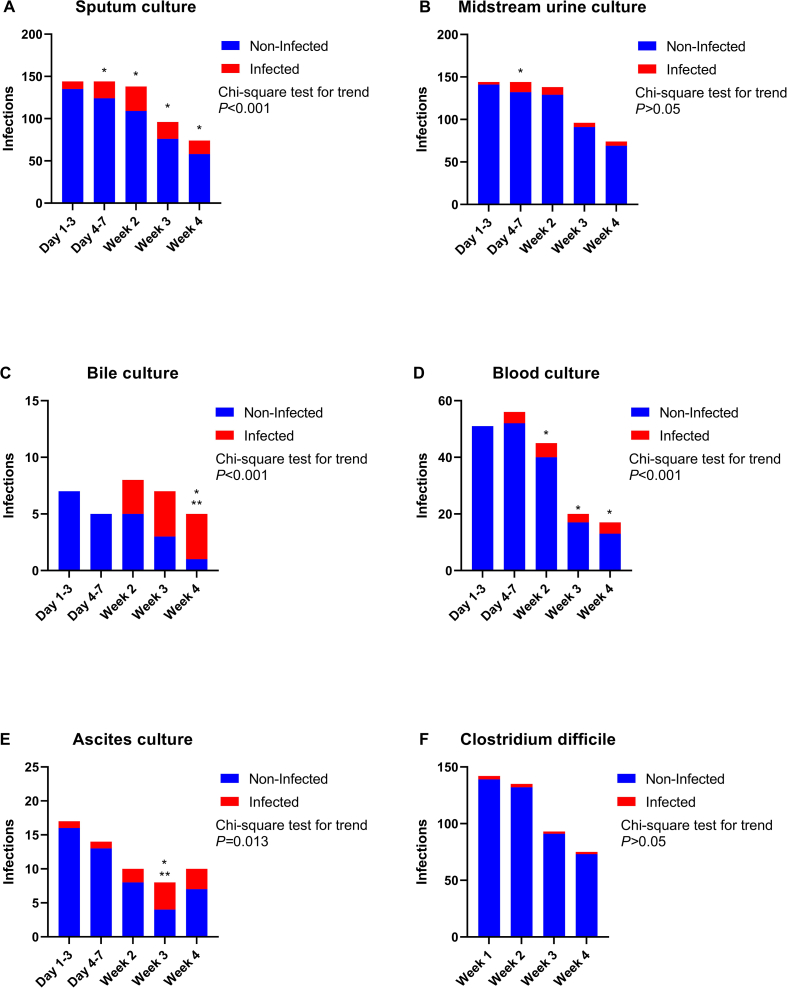
The changing trend of extrapancreatic infection in different sites. (A) sputum culture, (B) midstream urine culture, (C) bile culture, (D) blood culture, (E) ascites culture, (F) Clostridium difficile. *P < 0.05 compared to Day 1–3, **P < 0.05 compared to Day 4–7.

Among the 6 sites of extrapancreatic infection within 4 weeks after admission, the trend test for the proportion of infection was significantly different in sputum culture (Fig. 2A), bile culture (Fig. 2C), blood culture (Fig. 2D), and ascites (Fig. 2E). The positive rate of sputum culture was significantly higher in 4–7 days, which was statistically different from that in 1–3 days after admission. At the same time, the positive rates of blood culture, ascites culture, and bile culture in the second, third, and fourth weeks were significantly higher than those in the first to third day after admission (*P* < 0.05). The trend test for midstream urine culture (Fig. 2B) and Clostridium difficile (Fig. 2F) was negative.

### 3.4 Risk factor for extrapancreatic infection

Through binary logistic regression analysis, it was found that the main risk factor for respiratory tract infection was the duration of mechanical ventilation, the main risk factors for urinary system infection were gender, body mass index and Modified Marshall score, the main risk factors for bacteremia were duration of mechanical ventilation, blood urea nitrogen, diabetes mellitus and systolic blood pressure, the main risk factor for ascites infection was the duration of mechanical ventilation, and clostridium difficile infection was mainly related to mechanical ventilation and total bilirubin (Table 3).

**Table 3:** Binary logistic regression analysis of risk factors for extrapancreatic infection at different sites.

	Adjusted OR (95%CI)	*P* adjusted
Respiratory tract infection		
Duration of mechanical ventilation	1.283 (1.128–1.459)	<0.001∗
Urinary tract infection		
Gender	6.435 (1.986–20.847)	0.002∗
Body mass index	1.158 (1.024–1.310)	0.019∗
Modified Marshall score	1.421 (1.004–2.012)	0.048∗
Biliary tract infection		
None	/	/
Bacteremia		
Duration of mechanical ventilation	1.062 (1.012–1.114)	0.014∗
Blood urea nitrogen	1.196 (1.054–1.357)	0.005∗
Diabetes mellitus	7.458 (1.096–50.737)	0.040∗
Systolic blood pressure	1.037 (1.002–1.072)	0.037∗
Ascites infection		
Duration of mechanical ventilation	1.096 (1.019–1.178)	0.013∗
Clostridium difficile infection		
Mechanical ventilation	14.508 (2.775–75.834)	0.002∗
Total bilirubin	1.026 (1.003–1.049)	0.029∗

*P* adjusted: assessed by binary logistic regression; adjusted for age, gender, body mass index, etiology, hypertension, diabetes mellitus, white blood cell, C-reactive protein, procalcitonin, serum amylase, alanine aminotransferase, total bilirubin, blood urea nitrogen, serum creatinine, oxygenation index, systolic blood pressure, modified Marshall score, Bedside Index for Severity in Acute Pancreatitis score, Acute Physiology and Chronic Health EvaluationⅡ score, necrosis and length of hospital stay.

∗ *P* < 0.05 was considered statistically significant.

Infections of the respiratory tract were closely related to mechanical ventilation, while, infections of the respiratory tract were associated with longer duration of mechanical ventilation (Table 4).

**Table 4:** Analysis of the relationship between respiratory infections and mechanical ventilation in patients with patients with MSAP or SAP (n%; $\bar{x}$ ±s).

	Patients with respiratory infection	Patients without respiratory infection	*P* value
Mechanical ventilation	n = 40 23 (57.50%)	n = 104 12 (11.54%)	<0.001∗
Duration of mechanical ventilation (days)	n = 23 19.96 ± 18.61	n = 12 5.83 ± 3.10	0.014∗

∗ *P* < 0.05 was considered statistically significant.

### 3.5 Effect of extrapancreatic infection on prognosis

The impact of extrapancreatic infection on prognosis is listed in Table 5. With regard to pancreatic necrosis, patients with extrapancreatic infection had significantly higher rates of moderate and severe pancreatic necrosis (30–50% and greater than 50%) compared with patients without extrapancreatic infection. At the same time, the rate of laparotomy, length of hospital stay and in-hospital mortality were also significantly increased.

**Table 5:** The influence of extrapancreatic infection on prognosis in patients with MSAP or SAP (n%; $\bar{x}$ ±s).

	Extrapancreatic infection group (n = 58)	Non-extrapancreatic infection group (n = 86)	*P* value
Necrosis			
0–30%	39 (67.24%)	77 (89.53%)	0.001∗tbl5fnlowast
30–50%	10 (17.24%)	5 (5.81%)	0.028∗
>50%	9 (15.52%)	4 (4.65%)	0.026∗
Laparotomy	12 (20.69%)	0 (0.00%)	<0.001∗
Length of hospital stay (days)	47.05 ± 37.21	23.53 ± 12.87	<0.001∗
In-hospital mortality	9 (15.52%)	1 (1.16%)	0.001∗

∗ *P* < 0.05 was considered statistically significant.

## 4 Discussion

Until recent years, researchers have been concerned about extrapancreatic infection in hospital. The main finding of our study is that factors related to extrapancreatic infections were inflammatory factor as well as organ function damage. We found that C-reactive protein and procalcitonin in the extrapancreatic infection group were significantly higher than those in the non-infection group. And one systematic review [18] recommends using procalcitonin, rather than WBC or CRP, as a biomarker to guide antibiotic use. It is partially consistent with our research results. The main reason for the divergence is the different research objects. In this review, the classification of infection was mainly pancreatic infection or not, while our classification was extra-pancreatic infection. And we did not include patients with mild acute pancreatitis.

Concurrently, blood urea nitrogen, serum creatinine, oxygenation index, modified Marshall score, BISAP score and APACHE II score were also significantly increased in the infection group. Therefore, it is not surprising that our study also found that the risk factors of extrapancreatic infection included diabetes mellitus, systolic blood pressure, blood urea nitrogen, Modified Marshall score, and the duration of mechanical ventilation. The duration of mechanical ventilation were the main risk factors of respiratory tract infection. Alcohol can lead to the host’s defense against invading pathogens [19], but we have not found any correlation between alcohol etiology and extrapancreatic infection, which may be related to the small sample size.

### 4.1 Characteristics of extrapancreatic infection

In 144 patients, extrapancreatic infection accounted for 40.28%, which was much higher than the 15% previously reported in Rou et al. [9]. This may be related to the serious condition of the patients we selected. The patients we enrolled were MSAP or SAP patients with modified Marshall score ≥2 on admission. Respiratory tract (40.82%), urinary tract (24.49%) and bacteremia (11.22%) were the most common sites of extrapancreatic infection. The incidence rate of respiratory tract and urinary tract was consistent with the results of Rou et al. [9]. Brown et al. [11] pointed out that the most common manifestations of extrapancreatic infections in SAP patients were respiratory infections (9.2%) and bacteremia (8.4%).

Interestingly, the patients with infection in one site was 58.62%, and the remaining patients (41.38%) had multiple site infections. Therefore, patients suspected of having extrapancreatic infections should be excluded from other site infections. Gram-negative bacteria were the main pathogens in extrapancreatic infections. Among them, Acinetobacter baumannii, Candida albicans and Klebsiella pneumoniae were the most common pathogen, which was consistent with some research results [11,20]. Immunosuppression in AP patients may lead to excessive systemic inflammatory response syndrome, leading to intestinal or in vivo bacterial translocation and extrapancreatic infection [21].

Meanwhile, the multidrug resistance rate of acinetobacter baumannii, candida albicans and klebsiella pneumoniae found in extrapancreatic infections was 98.21%, 13.51% and 85.71%, respectively. Recently, several studies have shown that the proportion of multidrug resistant bacterial infections in AP patients has risen to 63% [22]. Mourad et al. [23] also reported that long-term use of antibiotics can lead to multi drug resistant bacterial and fungal infections, which is also related to long-term hospitalization and poor prognosis. This resistance may be related to the use of broad-spectrum antibiotics and the decline of autoimmunity in SAP patients. These MDR bacteria have become common pathogens of nosocomial infection through a variety of mechanisms, such as target alteration, drug inactivation, decreased cell permeability and enhanced efflux pump activity [22,24,25]. Therefore, the prevention and treatment of drug resistant bacterial infection should not be disregarded, and secondary infections should be closely monitored during hospital treatment and nursing, especially in SAP patients.

### 4.2 Extrapancreatic infection and the course of disease

Our new finding that the earliest infection occurs 4–7 days after admission, the positive rates of sputum culture, blood culture, ascites culture and bile culture in the second, third and fourth weeks were significantly higher than those in the first to third days after admission. The proportion of infection positive rates in various parts increased with the course of disease.

In the early stage, extrapancreatic infections may occur due to the suppression of immune function by systemic inflammatory response [26]. The causes of late extrapancreatic infection may be related to immunosuppression caused by long hospital stay or multiple operations, or secondary iatrogenic infection caused by deep venous catheter and long-term parenteral nutrition catheter [7,27]. Final, with the development of the disease, patients will continue to develop severe immunosuppressive inflammation [7].

### 4.3 Extrapancreatic infections and patient outcome

Consistent with previously published data [9], the occurrence of extrapancreatic infection was associated with the severity of pancreatitis, longer hospital stay, and increased in-hospital mortality. Moka et al. [20] noted that the total length of stay, ICU length of stay and mortality of SAP patients with extrapancreatic infection (pneumonia or bacteremia) were significantly higher than those without extrapancreatic infection. Secondary infection is an important factor affecting mortality in patients with SAP [1,3,4,6]. However, a meta-analysis showed that extrapancreatic infection was not associated with the predicted severity or mortality of acute pancreatitis [11]. Difference between the findings of the current and previous studies may be due to subject selection. Our study found that MSAP or SAP patients with extrapancreatic infection had poor prognosis. The main reason for the same results was that MSAP patients with transient organ dysfunction had a potential risk of evolving into SAP. Moran et al. [28] reported that early pancreatic infection is associated with increased mortality. Thus, both extrapancreatic and pancreatic infections are associated with increased mortality in patients with acute pancreatitis.

### 4.4 Limitation

However, our study also has some limitations. First, this is a single center retrospective study with a small sample size. Further study with larger sample size is necessary. In addition, due to the complexity of clinical treatment, there may be some deviation in the type and quantity of pathogenic bacteria. For example, antibiotic treatment can affect the resistance of pathogens. It needs to be divided into several subgroups for analysis through standardized antibiotic management. However, antibiotic treatment needs to be adjusted according to the patient’s condition and culture results, and the actual clinical practice is difficult. In the future, prospective studies are needed to verify the impact of initiating antibiotic therapy at different stages on the prognosis of pancreatitis. We used the revised Atlanta classification to determine the severity. Recently, the AI Severity Index (EASY SCORE) was released, and its application in retrospective prediction of AP severity was also proved by relevant research [29]. At present, all the scoring criteria are still not perfect, and further research is expected to confirm the accurate assessment of the severity of pancreatitis.

## 5 Conclusion

Our research has confirmed the need to prevent and monitor extrapancreatic infection in the early stage and provided a new basis for the prophylactic or therapeutic use of antibiotics in the early stage of acute pancreatitis. When AP patients have clinical symptoms of suspected infection, such as elevated inflammatory index or organ dysfunction or mechanical ventilation, priority should be given to the culture of extrapancreatic infection sites. When AP patients are accompanied with extrapancreatic infection, the risk of organ failure, pancreatic necrosis or multiple operations should be closely monitored. Therefore, early detection and the analysis of the risk factors for extrapancreatic infection in AP patients will help clinicians to use antibiotics effectively and reasonably, improve treatment efficacy, and reduce the related mortality of AP patients.

## Author contribution statement

Tongtian Ni: Conceived and designed the experiments; Performed the experiments; Analyzed and interpreted the data; Wrote the paper.

Yi Wen: Conceived and designed the experiments; Contributed reagents, materials, analysis tools or data.

Bing Zhao: Analyzed and interpreted the data.

Ning Ning: Performed the experiments; Analyzed and interpreted the data; Contributed reagents, materials, analysis tools or data.

Erzhen Chen: Contributed reagents, materials, analysis tools or data.

Enqiang Mao: Conceived and designed the experiments; Contributed reagents, materials, analysis tools or data; Wrote the paper.

Weijun Zhou: Conceived and designed the experiments; Wrote the paper.

## Funding statement

This work was supported by Clinical Research Project of Ruijin Hospital Affiliated to Shanghai Jiao Tong University School of Medicine, China [2018CR004] and 10.13039/100007219Shanghai Natural Science Foundation, China [21ZR1440400].

## Data availability statement

Data will be made available on request.

## Declaration of interest’s statement

The authors declare that they have no known competing financial interests or personal relationships that could have appeared to influence the work reported in this paper.
