# Supplementary material for: Characteristics and risk factors for extrapancreatic infection in patients with moderate or severe acute pancreatitis
Source: Heliyon. 2023 Jan 20;9(2):e13131. doi: 10.1016/j.heliyon.2023.e13131 (PMC9900262; doi:10.1016/j.heliyon.2023.e13131)
Supplement: Multimedia component 1 [file mmc1.docx]

Table S1 Sputum culture in patients with MSAP or SAP

|  | Pathogenic microorganisms | n (%) | Resistant to antibiotics, n (%) | | |
| --- | --- | --- | --- | --- | --- |
|  |  |  | S | R | MDR |
| Day 1-3  (n=144) | Acinetobacter baumannii  Acinetobacter junii  Klebsiella pneumoniae  Staphylococcus aureus  Candida albicans | 2 (22.22)  1 (11.11)  1 (11.11)  2 (22.22)  3 (33.33) | 0 (0.00)  0 (0.00)  0 (0.00)  0 (0.00)  3 (100.00) | 0 (0.00)  1 (100.00)  0 (0.00)  2 (100.00)  0 (0.00) | 2 (100.00)  0 (0.00)  1 (100.00)  0 (0.00)  0 (0.00) |
|  |  |  |  | | |
| Day 4-7  (n=144) | Acinetobacter baumannii  Klebsiella pneumoniae  Pseudomonas aeruginosa  Pseudomonas maltophilia  Escherichia coli  Staphylococcus aureus  Candida albicans | 7 (30.43)  5 (21.74)  1 (4.35)  1 (4.35)  1 (4.35)  3 (13.04)  5 (21.74) | 0 (0.00)  0 (0.00)  0 (0.00)  1 (100.00)  0 (0.00)  0 (0.00)  4 (80.00) | 0 (0.00)  2 (40.00)  0 (0.00)  0 (0.00)  0 (0.00)  1 (33.33)  1 (20.00) | 7 (100.00)  3 (60.00)  1 (100.00)  0 (0.00)  1 (100.00)  2 (66.67)  0 (0.00) |
|  |  |  |  | | |
| Week 2  (n=138) | Acinetobacter baumannii  Klebsiella pneumoniae  Pseudomonas maltophilia  Pseudomonas aeruginosa  Acinetobacter junii  Elizabethkingia meningoseptica  Staphylococcus aureus  Candida albicans  Aspergillus terreus | 14 (37.88)  6 (16.22)  4 (10.81)  3 (8.11)  1 (2.70)  1 (2.70)  4 (10.81)  3 (8.11)  1 (2.70) | 0 (0.00)  0 (0.00)  4 (100.00)  0 (0.00)  0 (0.00)  0 (0.00)  0 (0.00)  2 (66.67)  0 (0.00) | 1 (7.14)  2 (33.33)  0 (0.00)  0 (0.00)  1 (100.00)  0 (0.00)  2 (50.00)  1 (33.33)  1 (100.00) | 13 (92.84)  4 (66.67)  0 (0.00)  3 (100.00)  0 (0.00)  1 (100.00)  2 (50.00)  0 (0.00)  0 (0.00) |
|  |  |  |  | | |
| Week 3  (n=96) | Acinetobacter baumannii  Pseudomonas maltophilia  Klebsiella pneumoniae  Pseudomonas aeruginosa  Elizabethkingia meningoseptica  Staphylococcus aureus  Candida albicans  Candida parapsilosis  Aspergillus terreus | 12 (42.86)  4 (14.29)  3 (10.71)  1 (3.57)  1 (3.57)  2 (7.14)  3 (10.71)  1 (3.57)  1 (3.57) | 0 (0.00)  3 (75.00)  0 (0.00)  0 (0.00)  0 (0.00)  0 (0.00)  2 (66.67)  1 (100.00)  1 (100.00) | 0 (0.00)  1 (25.00)  0 (0.00)  0 (0.00)  0 (0.00)  0 (0.00)  0 (0.00)  0 (0.00)  0 (0.00) | 12 (100.00)  0 (0.00)  3 (100.00)  1 (100.00)  1 (100.00)  2 (100.00)  1 (33.33)  0 (0.00)  0 (0.00) |
|  |  |  |  | | |
| Week 4  (n=74) | Acinetobacter baumannii  Pseudomonas maltophilia  Pseudomonas aeruginosa  Klebsiella pneumoniae  Proteus mirabilis  Elizabethkingia meningoseptica  Staphylococcus aureus  Candida albicans  Candida parapsilosis  Candida krusei  Candida glabrata | 9 (39.13)  3 (13.04)  2 (8.70)  1 (4.35)  1 (4.35)  1 (4.35)  1 (4.35)  2 (8.70)  1 (4.35)  1 (4.35)  1 (4.35) | 0 (0.00)  2 (66.67)  0 (0.00)  0 (0.00)  0 (0.00)  0 (0.00)  0 (0.00)  1 (50.00)  1 (100.00)  1 (100.00)  1 (100.00) | 0 (0.00)  1 (33.33)  0 (0.00)  0 (0.00)  0 (0.00)  0 (0.00)  0 (0.00)  0 (0.00)  0 (0.00)  0 (0.00)  0 (0.00) | 9 (100.00)  0 (0.00)  2 (100.00)  1 (100.00)  1 (100.00)  1 (100.00)  1 (100.00)  1 (50.00)  0 (0.00)  0 (0.00)  0 (0.00) |

Note: S: sensitive to any antibiotic; R: resistant to <3 groups of antibiotics; MDR: Resistant to ≥3 groups of antibiotics.

Table S2 Midstream urine culture in patients with MSAP or SAP

|  | Pathogenic microorganisms | n (%) | Resistant to antibiotics, n (%) | | |
| --- | --- | --- | --- | --- | --- |
|  |  |  | S | R | MDR |
| Day 1-3  (n=144) | Candida albicans  Candida glabrata | 2 (66.67)  1 (33.33) | 1 (50.00)  1 (100.00) | 1 (50.00)  0 (0.00) | 0 (0.00)  0 (0.00) |
|  |  |  |  | | |
| Day 4-7  (n=144) | Escherichia coli  Acinetobacter baumannii  Klebsiella pneumoniae  Enterococcus faecium  Candida albicans  Candida glabrata  Candida tropicalis | 2 (15.38)  1 (7.69)  1 (7.69)  1 (7.69)  4 (30.77)  2 (15.38)  2 (15.38) | 0 (0.00)  0 (0.00)  0 (0.00)  0 (0.00)  1 (25.00)  2 (100.00)  2 (100.00) | 0 (0.00)  0 (0.00)  0 (0.00)  0 (0.00)  2 (50.00)  0 (0.00)  0 (0.00) | 2 (100.00)  1 (100.00)  1 (100.00)  1 (100.00)  1 (25.00)  0 (0.00)  0 (0.00) |
|  |  |  |  | | |
| Week 2  (n=138) | Acinetobacter baumannii  Enterococcus faecium  Candida albicans  Candida glabrata  Candida tropicalis | 2 (22.22)  2 (22.22)  3 (33.33)  1 (11.11)  1 (11.11) | 0 (0.00)  0 (0.00)  2 (66.67)  1 (100.00)  0 (0.00) | 0 (0.00)  0 (0.00)  1 (33.33)  0 (0.00)  1 (100.00) | 2 (100.00)  2 (100.00)  0 (0.00)  0 (0.00)  0 (0.00) |
|  |  |  |  | | |
| Week 3  (n=96) | Klebsiella pneumoniae  Acinetobacter baumannii  Enterococcus faecium  Candida albicans  Candida tropicalis | 2 (25.00)  1 (12.50)  1 (12.50)  3 (37.50)  1 (12.50) | 0 (0.00)  0 (0.00)  0 (0.00)  3 (100.00)  1 (100.00) | 0 (0.00)  0 (0.00)  0 (0.00)  0 (0.00)  0 (0.00) | 2 (100.00)  1 (100.00)  1 (100.00)  0 (0.00)  0 (0.00) |
|  |  |  |  | | |
| Week 4  (n=74) | Acinetobacter baumannii  Candida albicans  Candida tropicalis | 1 (20.00)  2 (40.00)  2 (40.00) | 0 (0.00)  2 (100.00)  1 (50.00) | 0 (0.00)  0 (0.00)  0 (0.00) | 1 (100.00)  0 (0.00)  1 (50.00) |

Note: S: sensitive to any antibiotic; R: resistant to <3 groups of antibiotics; MDR: Resistant to ≥3 groups of antibiotics.

Table S3 Bile culture in patients with MSAP or SAP

|  | Pathogenic microorganisms | n (%) | Resistant to antibiotics, n (%) | | |
| --- | --- | --- | --- | --- | --- |
|  |  |  | S | R | MDR |
| Day 1-3  (n=7) | None | 0 (0.00) | / | / | / |
|  |  |  |  | | |
| Day 4-7  (n=5) | None | 0 (0.00) | / | / | / |
|  |  |  |  | | |
| Week 2  (n=8) | Acinetobacter junii  Enterococcus faecium  Candida albicans | 1 (33.33)  1 (33.33)  1 (33.33) | 0 (0.00)  0 (0.00)  1 (100.00) | 0 (0.00)  0 (0.00)  0 (0.00) | 1 (100.00)  1 (100.00)  0 (0.00) |
|  |  |  |  | | |
| Week 3  (n=7) | Klebsiella pneumoniae  Acinetobacter junii  Pseudomonas maltophilia  Enterococcus faecium  Candida albicans | 1 (16.67)  1 (16.67)  1 (16.67)  1 (16.67)  2 (33.33) | 0 (0.00)  0 (0.00)  1 (100.00)  0 (0.00)  1 (50.00) | 0 (0.00)  0 (0.00)  0 (0.00)  0 (0.00)  0 (0.00) | 1 (100.00)  1 (100.00)  0 (0.00)  1 (100.00)  1 (50.00) |
|  |  |  |  | | |
| Week 4  (n=5) | Pseudomonas maltophilia  Klebsiella pneumoniae  Acinetobacter junii  Enterococcus faecium  Candida albicans | 2 (33.33)  1 (16.67)  1 (16.67)  1 (16.67)  1 (16.67) | 2 (100.00)  0 (0.00)  0 (0.00)  0 (0.00)  0 (0.00) | 0 (0.00)  0 (0.00)  0 (0.00)  0 (0.00)  0 (0.00) | 0 (0.00)  1 (100.00)  1 (100.00)  1 (100.00)  1 (100.00) |

Note: S: sensitive to any antibiotic; R: resistant to <3 groups of antibiotics; MDR: Resistant to ≥3 groups of antibiotics.

Table S4 Blood culture in patients with MSAP or SAP

|  | Pathogenic microorganisms | n (%) | Resistant to antibiotics, n (%) | | |
| --- | --- | --- | --- | --- | --- |
|  |  |  | S | R | MDR |
| Day 1-3  (n=51) | None | 0 (0.00) | / | / | / |
|  |  |  |  | | |
| Day 4-7  (n=56) | Pseudomonas maltophilia  Staphylococcus hemolyticus  Staphylococcus capitis | 1 (25.00)  2 (50.00)  1 (25.00) | 1 (100.00)  0 (0.00)  0 (0.00) | 0 (0.00)  0 (0.00)  1 (100.00) | 0 (0.00)  2 (100.00)  0 (0.00) |
|  |  |  |  | | |
| Week 2  (n=45) | Acinetobacter baumannii  Klebsiella pneumoniae  Pseudomonas maltophilia Bacillus megaterium  Candida albicans | 2 (33.33)  1 (16.67)  1 (16.67)  1 (16.67)  1 (16.67) | 0 (0.00)  0 (0.00)  1 (100.00)  1 (100.00)  1 (100.00) | 0 (0.00)  0 (0.00)  0 (0.00)  0 (0.00)  0 (0.00) | 2 (100.00)  1 (100.00)  0 (0.00)  0 (0.00)  0 (0.00) |
|  |  |  |  | | |
| Week 3  (n=20) | Klebsiella pneumoniae  Citrobacter koseri  Candida albicans | 1 (33.33)  1 (33.33)  1 (33.33) | 0 (0.00)  0 (0.00)  1 (100.00) | 0 (0.00)  0 (0.00)  0 (0.00) | 1 (100.00)  1 (100.00)  0 (0.00) |
|  |  |  |  | | |
| Week 4  (n=17) | Klebsiella pneumoniae  Acinetobacter baumannii  Staphylococcus hemolyticus | 2 (50.00)  1 (25.00)  1 (25.00) | 0 (0.00)  0 (0.00)  0 (0.00) | 0 (0.00)  0 (0.00)  0 (0.00) | 2 (100.00)  1 (100.00)  1 (100.00) |

Note: S: sensitive to any antibiotic; R: resistant to <3 groups of antibiotics; MDR: Resistant to ≥3 groups of antibiotics.

Table S5 Microbiologic findings in ascites in patients with MSAP or SAP

|  | Pathogenic microorganisms | n (%) | Resistant to antibiotics, n (%) | | |
| --- | --- | --- | --- | --- | --- |
|  |  |  | S | R | MDR |
| Day 1-3  (n=17) | Acinetobacter baumannii | 1 (100.00) | 0 (0.00) | 0 (0.00) | 1 (100.00) |
|  |  |  |  | | |
| Day 4-7  (n=14) | Klebsiella pneumoniae  Staphylococcus aureus | 1 (50.00)  1 (50.00) | 0 (0.00)  0 (0.00) | 0 (0.00)  1 (100.00) | 1 (100.00)  0 (0.00) |
|  |  |  |  | | |
| Week 2  (n=10) | Acinetobacter baumannii  Klebsiella pneumoniae | 1 (50.00)  1 (50.00) | 0 (0.00)  0 (0.00) | 0 (0.00)  0 (0.00) | 1 (100.00)  1 (100.00) |
|  |  |  |  | | |
| Week 3  (n=8) | Acinetobacter baumannii  Enterococcus faecium | 3 (75.00)  1 (25.00) | 0 (0.00)  0 (0.00) | 0 (0.00)  0 (0.00) | 3 (100.00)  1 (100.00) |
|  |  |  |  | | |
| Week 4  (n=10) | Acinetobacter baumannii  Klebsiella pneumoniae  Enterococcus faecium  Candida albicans | 1 (25.00)  1 (25.00)  1 (25.00)  1 (25.00) | 0 (0.00)  0 (0.00)  0 (0.00)  0 (0.00) | 0 (0.00)  0 (0.00)  0 (0.00)  1 (100.00) | 1 (100.00)  1 (100.00)  1 (100.00)  0 (0.00) |

Note: S: sensitive to any antibiotic; R: resistant to <3 groups of antibiotics; MDR: Resistant to ≥3 groups of antibiotics.

Table S6 Clostridium difficile in the stool in patients with MSAP or SAP

|  | Patients tested for Clostridium difficile | Patients infected with Clostridium difficile n (%) |
| --- | --- | --- |
|  |  |  |
| Week 1 | 142 | 3 (2.11) |
| Week 2 | 135 | 3 (2.22) |
| Week 3 | 93 | 2 (2.15) |
| Week 4 | 75 | 2 (2.67) |
